# Nest Survival and Failure in Ruffs Breeding on Grazed Coastal Meadows

**DOI:** 10.1002/ece3.73166

**Published:** 2026-03-31

**Authors:** Hanna Algora, Krisztina Kupán, Jelena Belojević, Veli‐Matti Pakanen, James D. M. Tolliver, Veronika A. Rohr‐Bender, Kari Koivula, Clemens Küpper

**Affiliations:** ^1^ Department of Ecology and Genetics University of Oulu Oulu Finland; ^2^ Research Group Behavioural Genetics and Evolutionary Ecology Max Planck Institute for Biological Intelligence Seewiesen Germany

**Keywords:** artificial incubation, cattle trampling, flooding, nest survival, predation, ruffs, wader conservation

## Abstract

Nest survival is a key determinant of birds' breeding success, particularly in ground‐breeding birds, where nest predation is a major cause of reproductive failure. Birds can maximise their nesting success by optimising aspects of the sociospatial environment of a nest, for example, by reducing the risk of predation or flooding. Previous research showed that ruff (*Calidris pugnax*) females nest closer to leks and to nests of conspecifics, and in some years, further from the shoreline and from meadow edges than expected by chance. However, whether and how social and environmental factors affect nest survival remains unclear. To fill this gap in knowledge, we analysed daily nest survival in a breeding population of ruffs, using nest locations from six breeding seasons. Following a Bayesian approach, we estimated daily nest survival rates to assess the impacts of social, spatial and temporal factors on ruff nest survival in three models focusing on distinct sources of mortality: (i) all sources of mortality (*Overall* model), (ii) all sources of mortality except for predation (*No‐predation* model) and (iii) only predation (*Predation* model). We also studied the effects of an intervention to protect nests at increased risk of failure by replacing their eggs with plastic dummies and artificially incubating the collected clutches. Overall, younger nests and those at intermediate distances to other nests had the highest daily nest survival, whereas laying date and distance to paths had a negative effect on daily nest survival in the *No‐predation* model. The use of dummy eggs reduced nest mortality of protected nests at strong risk of failure from flooding and cattle trampling, as well as the impact of predation. We highlight that identifying the sources of nest mortality is necessary to inform species and habitat management, ameliorate productivity and improve the recovery of endangered wader populations.

## 1 Introduction

Reproductive success is a key element sustaining population growth. Based on habitat selection theory, breeding birds should choose nesting habitats and locations that maximise their lifetime reproductive success (Clark and Shutler 1999; Hildén 1965). Preferred habitats should provide safety from predators, proximity to food resources and shelter from environmental hazards for both adults and their young (Clark and Shutler 1999; Cunningham et al. 2016; Hildén 1965; Martin and Roper 1988; Smith et al. 2007; With and Webb 1993).

Nest survival plays a crucial role in birds' breeding success, especially in ground‐breeding birds, where nest predation is the major cause of reproductive failure potentially leading to population decline (Douglas et al. 2023; Kentie et al. 2015; Laidlaw et al. 2017; Lima 2009; Macdonald and Bolton 2008; Magaña et al. 2010; Martin 1993b; Miller et al. 2007; Plard et al. 2020; Roos 2002; Sieving and Willson 1999). Therefore, reducing predation risk is considered a major criterion for nest‐site selection in birds (Forstmeier and Weiss 2004; Magaña et al. 2010; Martin 1993b; Miller et al. 2007). Birds can optimise aspects of the socio‐spatial environment of a nest to maximise nesting success and reduce the risk of predation by considering different aspects of the habitat's microrelief and microclimate, proximity to breeding neighbours for shared vigilance, clutch camouflage and other spatial features such as foraging locations and neighbouring habitats (Cunningham et al. 2016; Götmark and Andersson 1984; Gupta et al. 2022; Hancock et al. 2023; Lima 2009; Stoddard et al. 2016; Troscianko et al. 2016).

Proximity to resources may also affect nest predation risk. Nesting in resource‐rich areas may reduce predation risk during incubation by allowing incubating birds to reduce the amount of time that they stay away from their nests and offspring for foraging (Olsson and Bolin 2014). Nevertheless, foraging requirements do not always drive nest site selection (Smith et al. 2007), as birds also need to consider concealment from predators and predator detection. Open habitats such as saltmarshes and grasslands offer little‐to‐no cover for ground‐nesting birds, making it easier for visual predators to detect them. Therefore, birds must compromise by choosing a nest site that offers some concealment while avoiding high vegetation that could impede predator detection (Gómez‐Serrano and López‐López 2014). The spatial configuration of the macrohabitat may exacerbate nest predation through edge effects, which occur at the intersection of abruptly contrasting habitats and lead to increased predator abundance (Huhta et al. 2015; McCollin 1998; Sammalisto 1957). Furthermore, the presence of paths throughout the grassy habitat can provide travel routes for ground predators, making it easier for them to access nesting areas (Andrén 1995; McCollin 1998).

Ground‐nesting waders that breed in open agricultural landscapes are highly impacted by nest predation, which is considered to be one of the most prevalent threats to waders across Europe (Douglas et al. 2023; Ewing et al. 2023; Kentie et al. 2015; Kubelka et al. 2018; Laidlaw 2021; Pauliny et al. 2008; Rönkä et al. 2006; Roos et al. 2018). These regional trends reflect a global pattern, as wader populations have been globally declining over the last decades, specifically those in low‐lying shore habitats that are especially vulnerable to high predation rates (Cruz‐López et al. 2017; Kubelka et al. 2018; Laidlaw et al. 2015; Macdonald and Bolton 2008; Rönkä et al. 2006) and flooding (Bailey et al. 2017; Koivula et al. 2025). Habitat loss and degradation due to anthropogenic factors such as changes in agricultural practice and habitat fragmentation exacerbate both these risks (Piersma et al. 2016; Silva‐Monteiro et al. 2021; Studds et al. 2017). In Europe, a substantial number of waders breed in coastal meadows managed through cattle grazing subsidised by European agri‐environmental schemes. In managed meadow habitats their nests are at risk of failure due to predation, flooding and cow trampling, among other sources of mortality. In order to improve wader population recovery, it is often necessary to increase the breeding success by appropriate management, which is tailored to provide suitable habitat and, if necessary, decrease predation pressure (e.g., Douglas et al. 2023; Pearce‐Higgins et al. 2017).

Here we study the nest survival and sources of nest mortality in ruffs (*Calidris pugnax*), a lekking wader breeding on large and open shore meadows (Scheufler and Stiefel 1985; Thorup 2016; van Rhijn 1991). Previously, we showed that ruff females (called ‘reeves’) at the Bothnian Bay, Finland, tend to place their nests closer to leks and to nests of other reeves, and in some years, further from the shoreline and from meadow edges than expected by chance (Algora et al. 2025). However, it remained unclear whether and how the social and environmental factors that influence nest‐site location affect nest survival and predation risk. Identifying the sources of nest mortality is also relevant for other wader species breeding in these shore meadows as the sources of nest mortality are unlikely to be species‐specific.

We first analysed nest survival in a breeding population of ruffs and investigated how social (distance to leks and other ruff nests) and environmental spatial variables (distance to meadow edges, paths and shoreline) relate to daily survival probability of ruff nests. We have previously shown that ruff nests tend to be closer to other nests and leks than expected by chance, demonstrating that these are relevant social predictors of nest location (Algora et al. 2025). We predicted that nest survival might be highest at intermediate distances to the lek. Nesting close to leks may lead to increased male harassment, resulting in nest abandonment. At the same time, lekking males may indicate suitable habitat and act as sentinels, warning females of approaching predators (Phillips 1990). We hypothesised that survival would be highest at intermediate distances from other ruff nests as higher nest densities may attract predators (Frauendorf et al. 2022) whereas low densities might indicate less suitable nesting habitat. We also hypothesised that nests located close to paths (elevated disturbance and predation risk, Kaasiku et al. 2022), meadow edges (elevated high predation risk, Kaasiku et al. 2022) and the shoreline (elevated flooding risk, Koivula et al. 2025) would have lower survival.

Because ruff breeding populations are critically endangered, we tested the effectiveness of a management intervention, that is, clutch protection via egg replacement to improve nest survival. For this, we replaced clutches with plastic dummy eggs for a small sample of nests that were located in areas of intensive grazing or threatened from flooding by irregular storm surges (Koivula et al. 2025) and placed collected clutches in an incubator until hatching (‘artificial incubation’), after which we returned the chicks to the nests. We predicted that egg replacement would substantially improve nesting success as it would protect clutches in risky areas from the most important causes of mortality: predation, flooding and trampling.

## 2 Methods

### 2.1 Study Site

**FIGURE 1:**
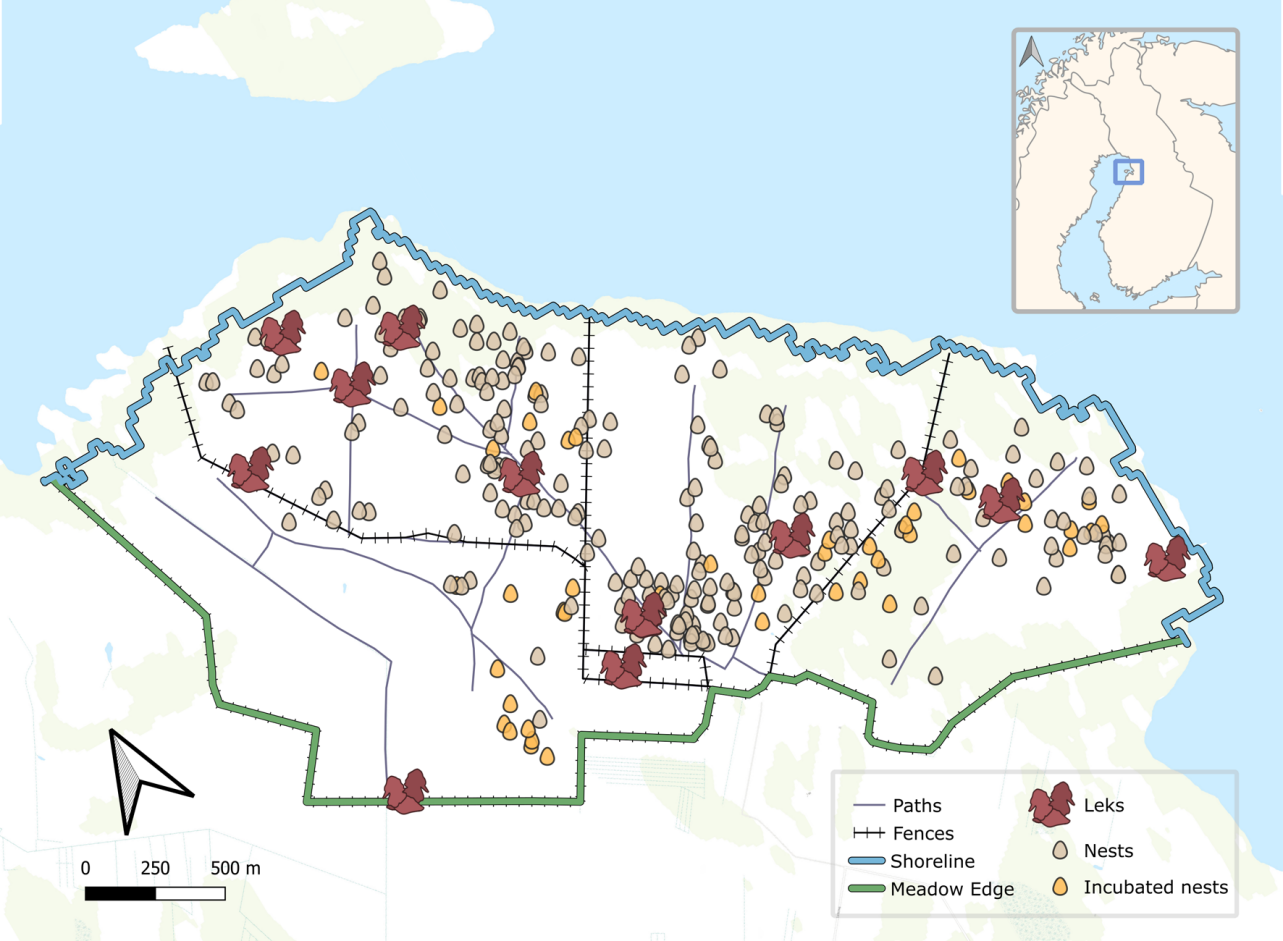
Study site (blue square in map inset) with nest and lek locations for years 2018–2023. Blue line represents the average shoreline, green line meadow edges and purple lines the paths crossing the meadow. Nests with clutches that were replaced and artificially incubated for over 24 h are shown in orange.

We studied survival of ruff nests located at Pitkänokka, Liminka Bay (ca. 64.86° N, 25.27° E, Figure 1), a large coastal meadow system (600 ha) at the Finnish coast of the Bothnian Bay. Because of isostatic land‐uplift and consequent succession, coastal marshes along the Baltic Sea in Finland consist of vegetation zones running parallel to the shoreline, with the areas close to the shoreline featuring low sward grasses, sedges and herbaceous plants that offer ideal nesting habitat for ground‐nesting waders (Devereux et al. 2004; Durant et al. 2008; Kaasiku et al. 2021; Siira 1970). Because of eutrophication and cessation of historical agricultural use, taller reeds ( *Phragmites australis* ) have overgrown parts of the formerly short grassy areas, making them less suitable for nesting of waders (Burnside et al. 2007; Kaasiku et al. 2021; Kose et al. 2021). Pitkänokka is surrounded by forest habitats and agricultural lands and the coastal meadows are managed through EU subsidised cattle grazing. The grazing is seasonally rotational with spring grazing starting in dedicated areas in the beginning of June and summer grazing starting in other areas after June 30.

### 2.2 Data Collection

#### 2.2.1 Lekking and Nesting Data

Ruffs breed in loose aggregations that feature lekking sites for male display in suitable nesting habitat (Algora et al. 2025). Male ruffs display on leks to attract females, but do not provide any resources other than sperm to females (Höglund and Alatalo 1995; Scheufler and Stiefel 1985; van Rhijn 1991). Reeves construct well‐hidden nests on the ground and are solely responsible for nesting and subsequent parental care. They incubate clutches for ca. 21 days and provide brood care for at least the first week after hatching of the chicks, but potentially until fledging (Andersen 1948; Giraldo‐Deck et al. 2022; Thorup 2016).

We collected data on nest fates at Pitkänokka from 2018 until 2023. We typically started nest searches in the second half of May, after the copulation peak at the leks. We carried out nest searches in groups of one to three people by walking across the meadows or rope‐dragging to flush incubating females. After locating a nest, we took GPS coordinates and identified a natural landmark or placed a natural object as a marker at 1–3 m in order to help with locating the nest again while minimising the chance that predators would learn to recognise our marks. We then counted, measured and floated the eggs to estimate the start of incubation (Liebezeit et al. 2007). We used a laying interval of 1.25 days per egg and an incubation period of 21 days to estimate the nest initiation dates. We checked the nests at least once before mid‐incubation to assess nest survival state and determine female ID. In the case of unringed females, we attempted to capture them after Day 7 (in 2018–19) or Day 14 (in 2020–2023) of incubation at the earliest, using walk‐in or snap traps. After Day 17, we visited nests every 2 days until we detected large cracks and then daily until the chicks hatched or the nest failed. For most nests, we captured and marked females and their young right after hatching. We marked females with a metal ring and either a combination of colour rings or, later, acrylic rings with an alphanumerical ID code.

We classified nests as ‘hatched’ when at least one chick had hatched. We considered nests to have ‘failed’ if we found clear evidence of failure or abandonment, or otherwise classified their fate as ‘unknown’. We further classified failed nests as ‘predated’ when the eggs had disappeared before hatching or when finding larger egg fragments, often bloodied, inside or close to the nest cup. We also considered nests to be predated if we found remains of the incubating reeves indicating predation, as this would inevitably lead to the failure of the nest.

In a few cases (*N* = 3 nests), the clutches disappeared near the expected hatching date, and we found small eggshell or membrane fragments in the nest cup indicating that at least one chick had hatched. Since we did not have information on the actual hatching date, we classified these nests as ‘escaped’, rather than hatched and censored them in the survival models (see below). We classified nests as ‘flooded’ when we found the nests submerged in water for several hours to days, with subsequent abandonment by the incubating reeve. We considered nests as ‘trampled’ when the eggs and/or nest cup were destroyed, and we detected clear signs of cow presence such as hoof marks or foraged and flattened grass. Finally, we considered a nest to have been abandoned when we found cold, dirty and/or unorganised eggs in the nest cup. We confirmed abandonment by positioning the eggs tip‐up in the nest cup. Since incubating reeves typically reposition their eggs upon returning to the nest, we classified nests as ‘abandoned’ if the eggs remained tip‐up at the following visit. In a few instances (*N* = 10 nests) we found signs of nest failure (e.g., damaged eggs) without having proof of predation or trampling, hence we classified these nests as ‘unknown dead’.

Due to high flooding and cattle trampling risks in some years, we collected high risk clutches, placed them in an incubator, and replaced them with dummy plastic eggs (‘artificially incubated clutches’). We placed the collected clutches in a Barotto incubator at a temperature of approximately 37°C and a humidity ranging from 40%–60% (with the optimal humidity at 50%), where the eggs were automatically rotated at regular intervals. We assessed embryo development every 5 to 7 days through candling, and we inspected eggs for cracks 4 days before the expected hatching date. Once a crack was found, we moved the entire clutch to a hatcher, providing a safe environment for hatching without egg turning, a temperature of approximately 37°C, and a humidity of 70%. We processed the chicks by taking biometric measurements and marking them with a metal ring, and returned them to the wild typically within 24 h of hatching (48 h in isolated cases). In the case of flooding risk, we replaced the eggs on the day in which the sea water level was predicted to rise 40 cm above the theoretical zero level. In the case of trampling risk, we replaced eggs with dummy eggs a few days prior to the release of cattle on the corresponding meadow. When the clutches with dummy eggs got destroyed or the female abandoned the nest, we cross‐fostered chicks that had hatched in the incubator to active nests of similar age. We incubated and cross‐fostered eggs in 2018, 2019, 2021 and 2022. In 2021, we only incubated the eggs from flood‐threatened nests for 1 day, the day of the flood. We did not consider these clutches as artificially incubated in the model, as they only housed dummy eggs for a short period of time, and hence their fate after our intervention would not be comparable to those nests that were artificially incubated until hatching. In 2020 we did not cross‐foster any chicks, and in 2023 we did not artificially incubate nor cross‐foster eggs nor chicks.

### 2.3 Data Preparation

During the study period, we found a total of 307 nests. From these, we excluded nests with relevant missing information such as missing dates (e.g., when the nest was found or floated) or mismatching spatial coordinates that were outside of our study area. After excluding these nests, we had 275 nests with complete data.

We used the exact or expected hatching date of a clutch to estimate lay date of the nest, from which date onwards we considered the nest to be active. We estimated the period the nest was active until hatching or nest failure. The modal value for nest activity of hatched nests was 26 days (range: 15–28 days). A larger deviation in the active period was often observed in nests that we had manipulated with cross‐fostering, artificial incubation or that had unhatched eggs (up to 33 days). Therefore, we only considered nest survival in our models for the activity period of 26 days after egg laying.

Because our data set was heterogeneous and included nests that we had manipulated to increase their survival, we ran the survival analyses with two different sets of models. First, the *Survival of all nests* models included the final fates of all nests. This, however, did not allow us to evaluate the full effect of flooding and cattle trampling as sources of mortality, since the replacement of natural eggs with dummies saved some nests from these fates. Therefore, in the second approach we excluded any data on nest fate after human intervention (*Survival of naturally incubated clutches*). For this, all nests that were incubated for any period of time were either censored or assigned as failed on the day of intervention. Specifically, we marked nests as failed on the day of intervention if their natural clutches had been collected due to flooding. Contrastingly, we censored nests of natural clutches collected because of cattle grazing on the day of intervention; that is, we considered the nest to continue being active until the day of intervention with the statistical model treating them alive at the last observation. We chose to censor these nests because, in contrast to flooding, cattle trampling presents an ongoing threat that makes it impossible to predict the exact day of nest failure.

### 2.4 Distances to Spatial Features

We used distance measurements to assess the effect of social (other nests and leks) and non‐social (paths, shoreline, meadow edges) spatial features on nest survival, estimated using *spatstat* in R (Baddeley and Turner 2005). We estimated the average shortest distance from a nest to other active nests, for which we first calculated daily shortest distance, that is, the distance from a nest to its closest active neighbour for every day when it was active. We then averaged the daily shortest distances for each nest into a mean shortest nest‐to‐nest distance (*distance to other nests*). We also estimated the distance from each nest to the centre of the closest lek (*distance to leks*), and to the closest point at the shoreline (*distance to shoreline*) and meadow edges (*distance to meadow edge*), respectively. We used satellite imagery for delimiting an average shoreline for the study period (see Algora et al. 2025) and we used the outer fence of the field site as meadow edges (Figure 1). Finally, we estimated the distance from each nest to the closest point of the closest path (*distance to paths*). We established meadow paths through visual inspection of the ESRI Satellite map layer in QGIS (v.3.22.5, QGIS Association 2023). Given that the selected paths were a multi‐line feature, we calculated distance to paths using *NNJOIN* in QGIS (v.3.22.5, QGIS Association 2023).

### 2.5 Statistical Analysis

**FIGURE A1:**
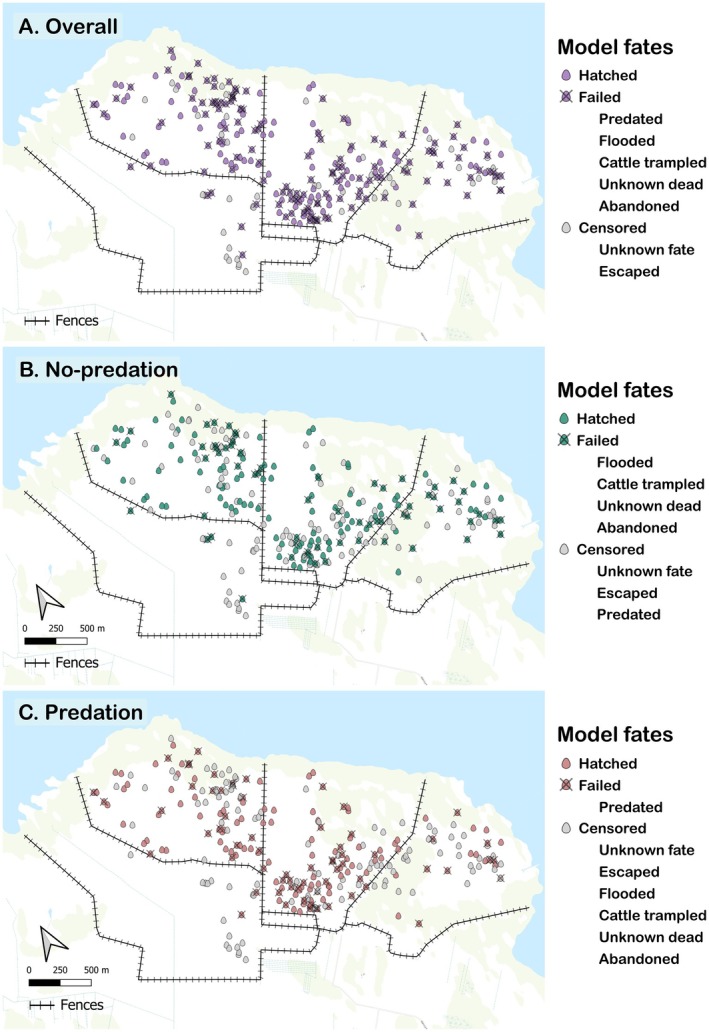
Map of 275 ruff nests with their corresponding fates used for the three models (‘Overall’, ‘No‐Predation’ and ‘Predation’) of nest survival. The models differ in their assignment of nests to ‘censored’ and ‘failed’ categories. This allowed us to disentangle spatial and temporal effects of nest predation, the main source of nest mortality, from effects of other sources of nest failure.

We tested the effect of the socio‐spatial and temporal variables on ruff nest survival by estimating daily nest survival rates (DSR) following a Bayesian approach (Korner‐Nievergelt et al. 2015). Nest failure in ruffs can have a number of reasons with potentially opposing effects on different sources of mortality. To disentangle the effects of potential predictors on predation, the main cause of nesting failure in our population (see Results), from those of other sources of nest mortality, we prepared three different models. Those models included (i) all sources of mortality (*Overall model*, Figure A1A), (ii) all sources of mortality but predation (*No‐predation model*, Figure A1B) and (iii) only predation (*Predation model*, Figure A1C). The models differed in the nests that were censored or had assigned an absolute fate (Figure A1). Whenever a nest had an unknown fate or perished due to sources of mortality that were not targeted by the specific model, we censored the nests on the date of failure. Censored nests are informative until the last day they were known to be active, but the model does not consider their fate as failed (Figure A1).

To estimate the effect of predictors on DSR, we prepared a binomial generalised mixed model with Bernoulli errors, using a Markov Chain Monte Carlo (MCMC) algorithm framework (Korner‐Nievergelt et al. 2015). We set up the model including fixed effects regarding spatial features (distance to shoreline, edges or paths), sociospatial features (distance to ruff nests or leks), temporal features (lay date and nest age) and management intervention (artificial incubation). To account for annual variation in DSRs, we added year as a random effect. Our sample may include nests of the same female, as our final data set included 275 nests from 110 females. The mother ID was unknown for less than half of the nests (118 nests); hence we did not include female ID as a random effect.

We expressed daily nest survival likelihood in our model as:

$$y_{\mathrm{DSR}}\left[i,t\right]\sim \text{bernoulli}\left(y_{\mathrm{DSR}}\left[i,t-1\right],\mathrm{Si},t\right);$$

$$\begin{array}{cc} \text{logit}\left(\mathrm{Si},t\right)\;=\; & \beta_{0}+\beta_{1}\text{distance}{\text{nests}}_{i}+\beta_{2}\text{distance}{\text{nests}}_{2i}\\ & +\beta_{3}\text{distance}{\text{leks}}_{i}+\beta_{4}\text{distance}{\text{leks}}_{2i}+\beta_{5}{\text{laydate}}_{i}\\ & +\beta_{6}\text{nest}\;{\mathrm{age}}_{t}+\beta_{7}{\text{incubation}}_{i}+\beta_{8}\text{distance}{\text{paths}}_{i}\\ & +\beta_{9}\text{distance}{\text{edge}}_{i}+\beta_{10}\text{distance}{\text{shore}}_{i}\\ & +\;\sigma \;\text{year}*u{\text{year}}_{i}; \end{array}$$

where $y_{\mathrm{DSR}}$ represents daily nest survival as a binary variable (‘1’ alive and ‘0’ dead) with dimensions i as the number of nests (*N* = 275) and t as the length of incubation including laying (0–26 days). S is the daily survival probability which relates to nest‐ and incubation day‐specific predictors of the model. Each fixed effect was centred and scaled around the yearly means (grouped data by year). Incubation was a binary variable (‘1’ artificially incubated for over 24 h, and “0” no artificial incubation for over 24 h). We used Gaussian priors (mean = 0, SD = 2) on the continuous fixed effects and on the random effects (mean = 0, SD = 1), and Cauchy priors on the categorical effects (mean = 0, SD = 2) and sigma parameters (mean = 0, SD = 5). Based on the three scenarios considering different sources of mortality, the model accounted for the nest becoming inactive in different ways. While the nest is active, the value for its fate on a given day is 1, and in the case of nest failure, the last day considered by the model was changed to 0. Conversely, for censored nests (Figure A1), the last value that the model considers is the last day the nest was active (i.e., survival was 1).

We ran the models using STAN through the R package *rstan* and inspected model convergence using the *shinystan* package (Stan Development Team 2024). We ran the models for 5000 iterations in 4 independent Markov chains, discarding 2500 iterations of burn‐in and using the remaining iterations to estimate posterior parameters. We checked for model convergence using the Gelman–Rubin statistics, with values under 1.10 indicating convergence (Brooks and Gelman 1998). We considered predictors to have a clear effect on DSR if the 95% Bayesian Credible Intervals (CrIs) of their mean did not overlap with 0.

To test for potential spatial autocorrelation between some of our predictors, we assessed the level of correlation between predictors using the R package *corrplot* (Wei and Simko 2021), and the Pearson correlation formula between paired samples to assess the significance of the correlation. Specifically, we assessed correlation between the predictors: (1) nest distances to shore and meadow edges; (2) nest distances to leks and shore; (3) nest distances to leks and meadow edges; (4) nest distances to leks and paths; (5) nest distances to shore and paths; and (6) nest distances to meadow edges and paths.

We repeated the same three sets of models for the dataset without nest fates after the intervention of artificial incubation (*Survival of naturally incubated clutches*), except that we excluded the fixed effect *Incubation*.

We used R version 4.3.3 (R Core Team 2023) for all data preparation and statistical analyses, and QGIS version 3.22.5 (QGIS Association 2023) for the map illustrations.

## 3 Results

### 3.1 Nests and Leks

We used a total of 275 nests monitored over the six‐year study period for our final models. Forty‐five of these clutches were collected and artificially incubated for more than 24 h and we noted a total of 12 lek locations (Figure 1) that were active in one or more study years.

### 3.2 Nest Fates of All Nests

Overall, 44.7% of the nests hatched (32%–60% across years, Table 1). Predation accounted for 54% of nest failures (44.4%–81.8% across years, Table 1). Nest abandonment (15.3% of failed nests, 4.5%–25% across years, Table 1) and cattle trampling (14.6% of failed nests, 2.8%–36.4% across years, Table 1) were the next important reasons for nest failure. Floods accounted for 8.8% (2.9%–22.2% across years, Table 1) of nest failure, and for the remaining 7.3% (3.7%–12.9% across years, Table 1) of failed nests, the reason for failure was unknown.

**TABLE 1:**
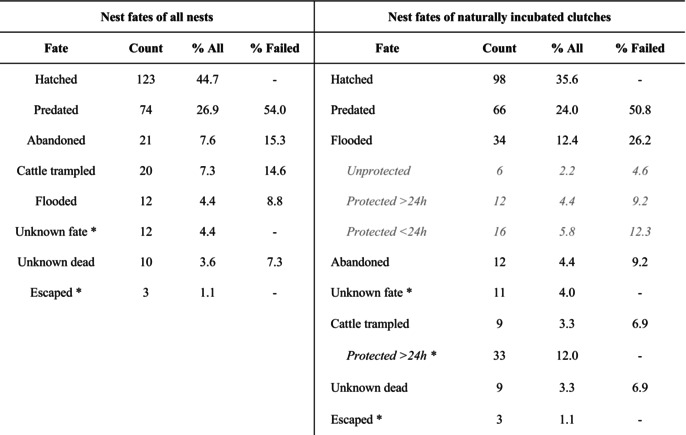
Nest fates for 2018–2023 based on the two sets of data analyses. ‘Nest fates of all nests’ includes the fates of nests also after artificial incubation used in ‘Survival of all nests’. ‘Nest fates of naturally incubated clutches’ includes the fates of nests until human intervention used in ‘Survival of naturally incubated clutches’. Nests that were protected against flooding were considered as failed due to flooding in the model.

^*^ Fates were censored in the models.

**FIGURE A2:**
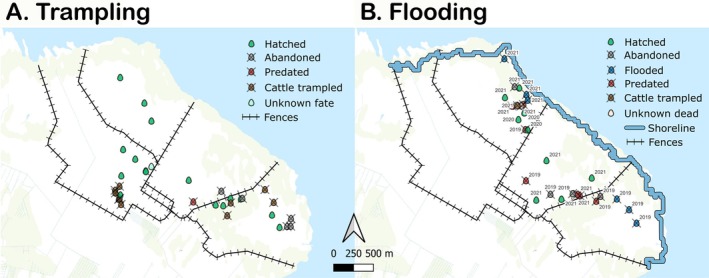
Location and fates of ruff nests that were selected for clutch replacement to protect the clutches from (A) cow trampling or (B) flooding. The left panel shows 33 nests whose clutches were artificially incubated because of the risk of trampling. Out of these, 16 clutches hatched, one clutch got predated and ten nest cups got trampled by cows leading to the female abandoning the nest. Five further nests were abandoned by the female, possibly because of disturbance by cows, and one had an unknown fate. The right panel shows all locations and fates of 28 clutches that were replaced and artificially incubated because they were threatened by flooding through a storm surge. Out of these, 16 clutches (all in 2021) were only incubated temporarily (< 24 h), while the rest were incubated until the chicks hatched. Of all these, six nests failed because of flooding, seven failed because of predation, four nests were abandoned, one nest was trampled by cows, and one failed with an unknown cause (‘unknown dead’). The remaining nine nests survived until hatching.

We artificially incubated clutches from 45 nests for more than 24 h because of the risk of flooding or trampling by cows. Of the 12 nests at risk of flooding, three hatched, three failed due to the flood despite our intervention, three failed because of predation, and three were abandoned later. Of the 33 nests at risk of trampling by cows, 16 clutches survived until hatching of the chicks, one clutch had an unknown fate, one clutch's dummy eggs were predated, and 10 nest cups got trampled, so that the reeve abandoned the nest (Figure A2). Five further nests were abandoned by the female likely because of disturbance by cows.

### 3.3 Nest Fates of Naturally Incubated Clutches

We included 275 nests in the *Survival of naturally incubated clutches* analysis, out of which 214 nests had no intervention. Less than half of the nests hatched (35.6%, 16%–54.8% across years, Table 1), and predation was the highest source of mortality, accounting for 50.8% (31.3%–81.8% across years, Table 1) of failed nests. Flooding corresponded to 26.2% (0%–53% across years, Table 1) of the failed nests: 4.6% perished without protection, and 21.5% were protected but would have died due to the flooding events (9.2% protected for more than 24 h, and 12.3% protected for less than 24 h, Table 1). Out of the remaining failed nests, 9.2% (5.3%–19% across years, Table 1) were abandoned, 6.9% (2.9%–26.3% across years, Table 1) were trampled by cattle, and another 6.9% (4.8%–9.4% across years, Table 1) died from unknown causes.

### 3.4 Nest Distances to Spatial and Socio‐Spatial Features

**FIGURE A3:**
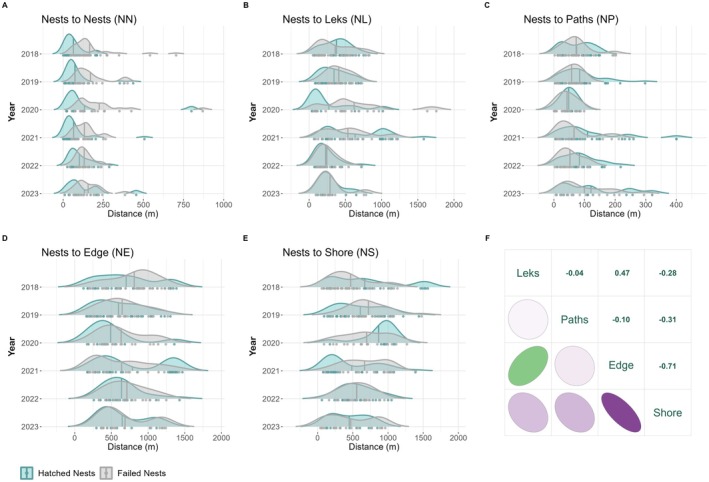
(A–E) Distributions of distances from nests to spatial features for hatched and failed nests per year, in turquoise and grey, respectively. Vertical lines show the average distance in a year, dots represent raw data. (F) Spatial correlation matrix between leks, meadow edges, shore and paths to nest distances.

Ruff nests were located on average at 121.2 m from other ruff nests (0 to 869.5 m, Figure A3A), and an average of 408.3 m away from leks (37.8 to 1755.8 m, Figure A3B). Nests were located on average at 78.7 m from meadow paths (0.5 to 400.2 m, Figure A3C), 690.6 m from meadow edges (120.4 and 1467.4 m, Figure A3D) and 580 m from the shoreline (39.3 and 1565.7, Figure A3E).

The distances from nests to meadow edges and from nests to the shoreline were negatively correlated with each other (Pearson's correlation coefficient: −0.71, *p* < 0.01, Figure A3F), indicating that a substantial number of nests were located at the part of the field site where the shoreline and meadow edges are directly opposing each other. Distance from nests to leks showed a weak but statistically clear negative correlation with the distance from nests to the shoreline (Pearson's correlation coefficient for shoreline: −0.28, *p* < 0.01, Figure A3F), while the distances from nests to leks and nests to meadow edges were weakly positively correlated with each other (Pearson's correlation coefficient: 0.47, *p* < 0.01, Figure A3F). Hence, nest to lek distances were somewhat dependent on nest to shoreline or nest to edge distances. There was no clear relationship between the distances from nests to paths and the distances from nests to leks and meadow edges (Pearson's correlation coefficient for leks: −0.04, *p* > 0.01; Pearson's correlation coefficient for meadow edges: −0.10, *p* > 0.01; Figure A3F).

### 3.5 Daily Nest Survival

#### 3.5.1 Survival of All Nests

**FIGURE 2:**
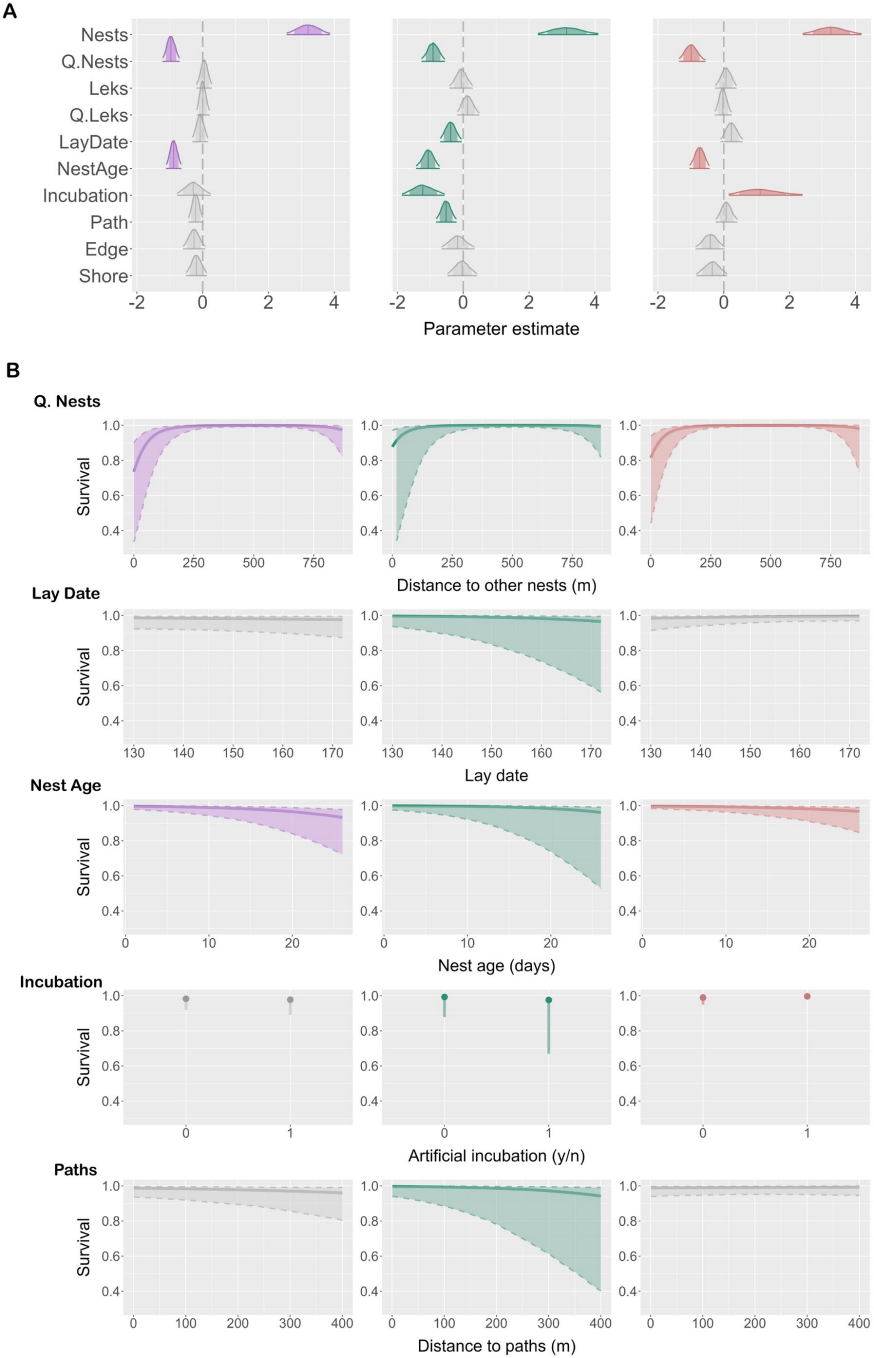
Predictors of daily nest survival probability for ‘Survival of all nests’ when all other predictors are kept at the mean. We ran three models that each accounted for different sources of mortality: Overall model (in purple), No‐predation model (in green) and Predation model (in red). (A) Summary of the posterior distributions of the fixed model predictors. The vertical lines represent the estimate means, the outer lines the 95% credible intervals (CrIs), and the shaded areas the 80% CrIs. Predictors with 95% CrIs that do not overlap with zero have a clear effect on nest survival (shown in purple, green and red for the respective models). Predictors shown in grey have 95% CrIs that overlap with zero. (B) Details for the fixed effects that had an effect on nest survival in either of the three models: Quadratic distance to other nests, lay date, nest age, incubation and distance to paths, showing the unscaled raw data for clarity. The continuous lines represent the mean estimates and the dashed lines the 95% CrIs.

We modelled daily nest survival for ruffs considering three scenarios: (1) the *Overall* model (‘Ov’), (2) the *No‐predation* model (‘NoP’) and (3) the *Predation* model (‘P’). The distance from nests to the closest nests had a negative quadratic effect (Figure 2, Table 2, mean_Ov_ = −0.96, 95% CrI_Ov_ = [−1.21, −0.71]; mean_NoP_ = −0.92, 95% CrI_NoP_ = [−1.26, −0.57]; mean_P_ = −0.99, 95% CrI_P_ = [−1.35, −0.57]) and a positive linear effect (Figure 2, Table 2, mean_Ov_ = 3.20, 95% CrI_Ov_ = [2.57, 3.86]; mean_NoP_ = 3.15, 95% CrI_NoP_ = [2.28, 4.09]; mean_P_ = 3.27, 95% CrI_P_ = [2.41, 4.18]) on the daily survival probability in all three models, meaning that daily nest survival was highest at intermediate nest distances, with mortality having a stronger effect on shorter than longer distances (Figure 2B). Similarly, nest age also had a negative effect on survival in all three models (Figure 2, Table 2, mean_Ov_ = −0.88, 95% CrI_Ov_ = [−1.1, −0.66]; mean_NoP_ = −1.07, 95% CrI_NoP_ = [−1.42, −0.73]; mean_P_ = −0.74, 95% CrI_P_ = [−1.03, −0.45]), meaning that daily nest survival decreased with age (Figure 2B). Distance to the meadow edges and shoreline had no clear effect on daily nest survival, although they showed a negative trend in the *Predation* model (Figure 2, Table 2, mean_P_ = −0.41, 95% CrI_P_ = [−0.84, 0.01] for meadow edges; mean_P_ = −0.36, 95% CrI_P_ = [−0.82, 0.09] for shoreline). Distance to leks had no effect on daily nest survival (Figure 2, Table 2).

**TABLE 2:** Model results for ‘Survival of all nests’. We ran three models that each accounted for different sources of mortality: Overall model, No‐predation model and Predation model. Variables for which the 95% credible intervals (CrIs) of the mean did not overlap with zero are shown in bold and highlighted in grey.

Overall model				No‐predation model				Predation model			
Variable	Mean	SD	95% CrI	Variable	Mean	SD	95% CrI	Variable	Mean	SD	95% CrI
**Nests**	**3.20**	**0.33**	**[2.57, 3.86]**	**Nests**	**3.15**	**0.46**	**[2.28, 4.09]**	**Nests**	**3.27**	**0.45**	**[2.41, 4.18]**
**Q.Nests**	**−0.96**	**0.13**	**[−1.21, −0.71]**	**Q.Nests**	**−0.92**	**0.18**	**[−1.26, −0.57]**	**Q.Nests**	**−0.99**	**0.20**	**[−1.35, −0.57]**
Leks	0.05	0.11	[−0.18, 0.27]	Leks	−0.06	0.17	[−0.41, 0.28]	Leks	0.07	0.16	[−0.25, 0.38]
Q.Leks	0.01	0.10	[−0.17, 0.2]	Q.Leks	0.14	0.17	[−0.17, 0.48]	Q.Leks	−0.03	0.12	[−0.26, 0.23]
LayDate	−0.08	0.12	[−0.3, 0.15]	**LayDate**	**−0.38**	**0.16**	**[−0.69, −0.06]**	LayDate	0.23	0.17	[−0.09, 0.56]
**NestAge**	**−0.88**	**0.11**	**[−1.1, −0.66]**	**NestAge**	**−1.07**	**0.17**	**[−1.42, −0.73]**	**NestAge**	**−0.74**	**0.15**	**[−1.03, −0.45]**
Incubation	−0.28	0.26	[−0.77, 0.23]	**Incubation**	**−1.22**	**0.33**	**[−1.85, −0.58]**	**Incubation**	**1.15**	**0.57**	**[0.16, 2.38]**
Path	−0.21	0.11	[−0.43, 0.01]	**Path**	**−0.52**	**0.15**	**[−0.82, −0.22]**	Path	0.08	0.16	[−0.24, 0.4]
Edges	−0.26	0.16	[−0.58, 0.06]	Edges	−0.17	0.25	[−0.65, 0.33]	Edges	−0.41	0.22	[−0.84, 0.01]
Shore	−0.19	0.16	[−0.5, 0.12]	Shore	−0.03	0.22	[−0.47, 0.41]	Shore	−0.36	0.23	[−0.82, 0.09]
**Intercept**	**4.00**	**0.69**	**[2.42, 5.17]**	**Intercept**	**4.76**	**1.08**	**[1.98, 6.36]**	**Intercept**	**4.52**	**0.69**	**[2.92, 5.73]**
**Sigma Year**	**1.53**	**0.62**	**[1.01, 3.19]**	**Sigma Year**	**2.17**	**1.19**	**[1.05, 5.46]**	**Sigma Year**	**1.46**	**0.59**	**[1.01, 3.11]**
Year 2018	0.36	0.41	[−0.46, 1.15]	Year 2018	0.40	0.42	[−0.43, 1.21]	Year 2018	0.38	0.44	[−0.5, 1.23]
Year 2019	−0.56	0.49	[−1.55, 0.37]	Year 2019	−0.50	0.56	[−1.62, 0.51]	Year 2019	−0.41	0.49	[−1.37, 0.53]
**Year 2020**	**0.98**	**0.47**	**[0.09, 1.93]**	**Year 2020**	**1.35**	**0.54**	**[0.35, 2.51]**	Year 2020	0.72	0.48	[−0.22, 1.67]
Year 2021	−0.11	0.44	[−0.98, 0.73]	Year 2021	−0.20	0.49	[−1.18, 0.7]	Year 2021	0.17	0.45	[−0.74, 1.03]
Year 2022	0.34	0.42	[−0.49, 1.15]	Year 2022	0.35	0.42	[−0.52, 1.15]	Year 2022	0.44	0.45	[−0.45, 1.33]
Year 2023	0.52	0.43	[−0.34, 1.36]	**Year 2023**	**0.92**	**0.50**	**[0, 1.97]**	Year 2023	0.29	0.46	[−0.64, 1.17]

Lay date and the distance from nests to paths had a negative effect on daily nest survival in the *No‐predation* model (Figure 2, Table 2, mean_NoP_ = −0.38, 95% CrI_NoP_ = [−0.69, −0.06]; mean_NoP_ = −0.52, 95% CrI_NoP_ = [−0.82, −0.22], for lay date and distance to paths, respectively). Therefore, mortality from reasons other than predation was higher at the end of the breeding season and further away from paths (Figure 2B), but there was no clear effect for these variables on daily nest survival in the other two models. Finally, artificial incubation had opposing effects on survival in the *No‐predation* and *Predation* model. Nests with artificially incubated clutches survived better than nests with naturally incubated clutches in the *Predation* model, whereas they had higher mortality than those with naturally incubated clutches in the *No‐predation* model (Figure 2, Table 2, mean_P_ = 1.15, 95% CrI_P_ = [0.16, 2.38]; mean_NoP_ = −1.22, 95% CrI_NoP_ = [−1.85, −0.58]). In contrast, there was no effect of artificial incubation in the *Overall* model (mean_Ov_ = −0.28, 95% CrI_Ov_ = [−0.77, 0.23]). There was a significant difference in daily nest survival between years in all three models (Table 2, mean_Ov_ = 1.53, 95% CrI_Ov_ = [1.01, 3.19]; mean_NoP_ = 2.17, 95% CrI_NoP_ = [1.05, 5.46]; mean_P_ = 1.46, 95% CrI_P_ = [1.01, 3.11]) suggesting an annual variation in daily nest survival rates.

**FIGURE 3:**
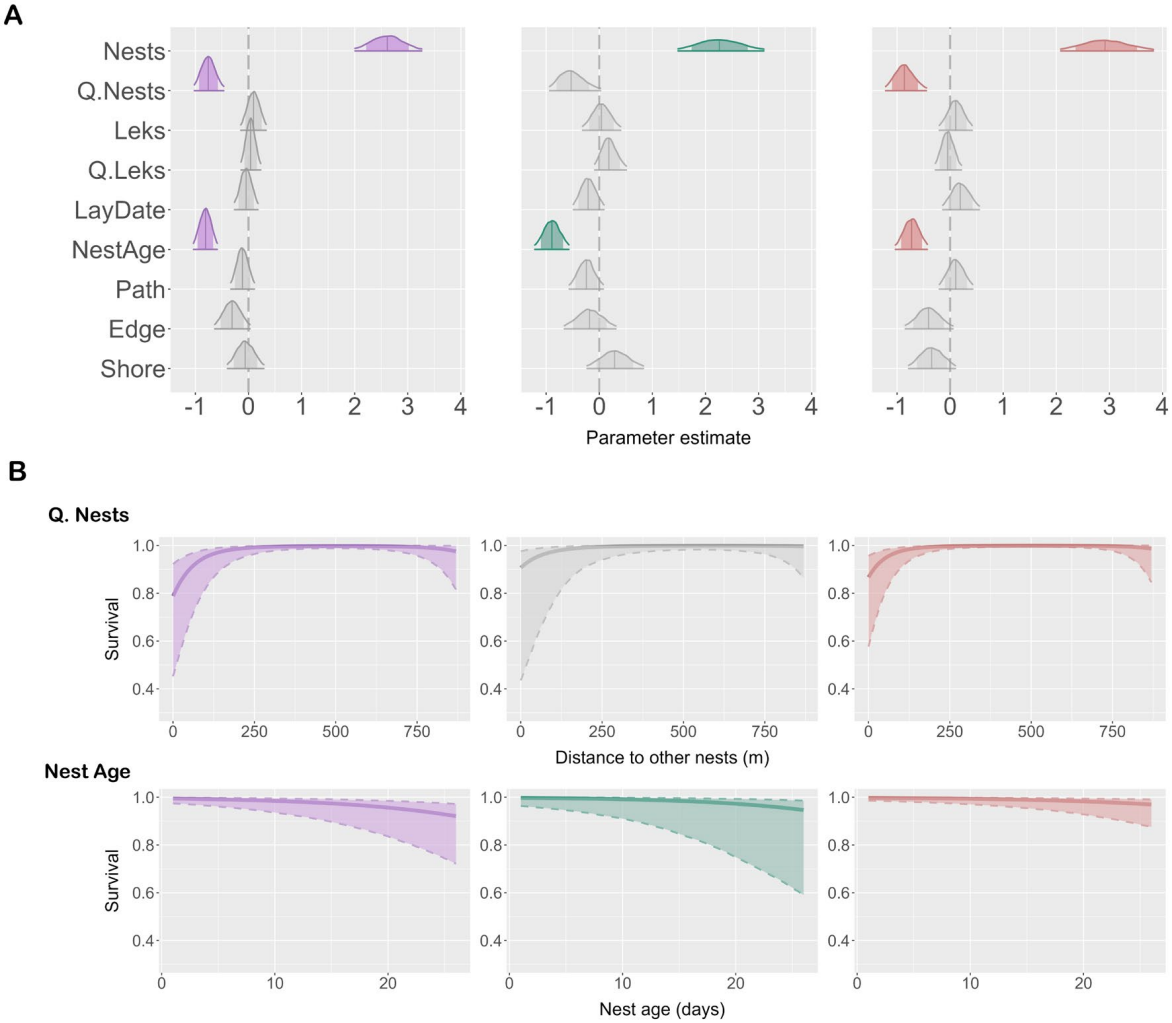
Predictors of daily nest survival probability for ‘Survival of naturally incubated clutches’ when all other predictors are kept at the mean. We ran three models that each accounted for different sources of mortality: Overall model (in purple), No‐predation model (in green) and Predation model (in red). (A) Summary of the posterior distributions of the fixed model predictors. The vertical lines represent the estimate means, the outer lines the 95% credible intervals (CrIs), and the shaded areas the 80% CrIs. Predictors with 95% CrIs that do not overlap with zero have a clear effect on nest survival (shown in purple, green and red for the respective models). Predictors shown in grey have 95% CrIs that overlap with zero. (B) Details for the fixed effects that had an effect on nest survival in either of the three models: Quadratic distance to other nests and nest age, showing the unscaled raw data for clarity. The continuous lines represent the mean estimates and the dashed lines the 95% CrIs.

*Survival of naturally incubated clutches*. The distance from nests to other nests had a clear negative quadratic effect on the daily survival probability in only the *Overall* and *Predation* models, and there was a trend in the same direction in the *No‐predation* model (Figure 3, Table 3, mean_Ov_ = −0.75, 95% CrI_Ov_ = [−1.02, −0.46]; mean_NoP_ = −0.51, 95% CrI_NoP_ = [−0.93, 0.02]; mean_P_ = −0.85, 95% CrI_P_ = [−1.22, −0.44]), meaning that daily nest survival was highest at intermediate nest distances (Figure 3B). Similarly to the data set considering fates of all nests, nest age also had a negative effect on daily nest survival in all three models for naturally incubated clutches (Figure 3, Table 3, mean_Ov_ = −0.81, 95% CrI_Ov_ = [−1.04, −0.59]; mean_NoP_ = −0.89, 95% CrI_NoP_ = [−1.21, −0.57]; mean_P_ = −0.73, 95% CrI_P_ = [−1.03, −0.43]), meaning that young nests had a higher daily survival probability than nests that were more advanced in the incubation stage (Figure 3B). Year had a clear effect on nest survival in all three models (Table 3, mean_Ov_ = 1.46, 95% CrI_Ov_ = [1.01, 3.01]; mean_NoP_ = 1.91, 95% CrI_NoP_ = [1.03, 4.59]; mean_P_ = 1.40, 95% CrI_P_ = [1.01, 2.85]), suggesting an annual variation in daily nest survival rates. Distance to edges and distance to the shoreline had no clear effect on nest survival, although both showed a negative trend in the *Predation* model (Figure 3, Table 3, mean_P_ = −0.4, 95% CrI_P_ = [−0.84, 0.06] for meadow edges; mean_P_ = −0.34, 95% CrI_P_ = [−0.78, 0.1] for shoreline), and distance to the shoreline showed a weak positive trend in the *No‐predation* model (Figure 3, Table 3, mean_NoP_ = 0.29, 95% CrI_NoP_ = [−0.23, 0.83]). Finally, distance to leks had no effect on daily nest survival (Figure 3, Table 3).

**TABLE 3:** Model results for ‘Survival of naturally incubated clutches’. We ran three models that each accounted for different sources of mortality: Overall model, No‐predation model and Predation model. Variables for which the 95% credible intervals (CrIs) of the mean did not overlap with zero are shown in bold and highlighted in grey.

Overall model				No‐predation model				Predation model			
Variable	Mean	SD	95% CrI	Variable	Mean	SD	95% CrI	Variable	Mean	SD	95% CrI
**Nests**	**2.61**	**0.32**	**[2, 3.27]**	**Nests**	**2.27**	**0.42**	**[1.48, 3.1]**	**Nests**	**2.93**	**0.45**	**[2.08, 3.83]**
**Q.Nests**	**−0.75**	**0.14**	**[−1.02, −0.46]**	Q.Nests	−0.51	0.25	[−0.93, 0.02]	**Q.Nests**	**−0.85**	**0.20**	**[−1.22, −0.44]**
Leks	0.10	0.12	[−0.15, 0.33]	Leks	0.04	0.18	[−0.32, 0.4]	Leks	0.11	0.16	[−0.21, 0.42]
Q.Leks	0.04	0.09	[−0.14, 0.23]	Q.Leks	0.19	0.15	[−0.09, 0.51]	Q.Leks	−0.04	0.13	[−0.28, 0.22]
LayDate	−0.04	0.11	[−0.26, 0.18]	LayDate	−0.21	0.15	[−0.49, 0.09]	LayDate	0.20	0.18	[−0.14, 0.55]
**NestAge**	**−0.81**	**0.12**	**[−1.04, −0.59]**	**NestAge**	**−0.89**	**0.16**	**[−1.21, −0.57]**	**NestAge**	**−0.73**	**0.15**	**[−1.03, −0.43]**
Path	−0.11	0.11	[−0.33, 0.11]	Path	−0.24	0.16	[−0.57, 0.08]	Path	0.11	0.16	[−0.2, 0.43]
Edges	−0.31	0.17	[−0.64, 0.03]	Edges	−0.18	0.25	[−0.66, 0.31]	Edges	−0.40	0.23	[−0.84, 0.06]
Shore	−0.06	0.17	[−0.4, 0.29]	Shore	0.29	0.27	[−0.23, 0.83]	Shore	−0.34	0.23	[−0.78, 0.1]
**Intercept**	**3.73**	**0.64**	**[2.32, 4.84]**	**Intercept**	**4.19**	**0.98**	**[1.91, 5.66]**	**Intercept**	**4.60**	**0.65**	**[3.17, 5.73]**
**Sigma Year**	**1.46**	**0.56**	**[1.01, 3.01]**	**Sigma Year**	**1.91**	**1.08**	**[1.03, 4.59]**	**Sigma Year**	**1.40**	**0.51**	**[1.01, 2.85]**
Year 2018	0.55	0.42	[−0.27, 1.38]	Year 2018	0.76	0.45	[−0.11, 1.66]	Year 2018	0.35	0.44	[−0.53, 1.2]
Year 2019	−0.49	0.46	[−1.39, 0.39]	Year 2019	−0.57	0.54	[−1.64, 0.46]	Year 2019	−0.16	0.47	[−1.1, 0.74]
Year 2020	0.65	0.44	[−0.21, 1.52]	Year 2020	0.63	0.47	[−0.28, 1.59]	Year 2020	0.62	0.47	[−0.29, 1.57]
Year 2021	−0.27	0.44	[−1.15, 0.57]	Year 2021	−0.47	0.52	[−1.51, 0.52]	Year 2021	0.19	0.46	[−0.74, 1.08]
Year 2022	0.43	0.42	[−0.38, 1.25]	Year 2022	0.52	0.44	[−0.34, 1.39]	Year 2022	0.37	0.45	[−0.5, 1.25]
Year 2023	0.56	0.44	[−0.29, 1.43]	**Year 2023**	**1.07**	**0.52**	**[0.11, 2.18]**	Year 2023	0.19	0.47	[−0.73, 1.08]

## 4 Discussion

Understanding the factors that govern nesting success in meadow‐breeding waders can provide important cues about population viability and guide conservation management. We examined the effects of spatial, social and temporal variables on daily nest survival in ruffs that breed on grazed coastal meadows at the Baltic Sea. Daily nest survival declined with nest age, and nests with intermediate distances to other nests had the highest probabilities of daily survival. Collecting and artificially incubating high‐risk clutches reduced their predation mortality although many of those clutches still failed because of other reasons. Our results are consistent with complex relationships between nest survival and spatial, social and temporal variables and highlight the need to understand the different components affecting survival both independently and holistically.

### 4.1 Overall Nest Survival

Considering all sources of nest mortality simultaneously (*Overall* model), we found that only nest‐to‐nest distance and nest age had a clear effect on daily nest survival. These two predictors were also influential in the *Predation* and *No‐predation* models. In contrast, *No‐predation* and *Predation* models showed opposing effects of lay date, distance to paths, distance to the shoreline and artificial incubation, which then cancelled each other out in the *Overall* model. This points to potential trade‐offs affecting nest survival such as between the risk of flooding and predation, which can ultimately influence optimal locations and timing of breeding by narrowing the availability of favourable breeding conditions. It also highlights the need to study the different sources of nest mortality independently.

### 4.2 Drivers of Nest Predation

Nest predation is one of the major causes of nest failure in ground‐breeding and open‐nesting birds (e.g., Macdonald and Bolton 2008; Martin 1993b, 1993a; Roos et al. 2018). Nevertheless, habitat and nest‐site choices can help to reduce predation risks (Kristan III et al. 2007; Pass et al. 2019; Söderström 2001). Our finding that ruff nests survived better at intermediate distances from other nests suggests that there is a trade‐off between the costs and benefits of nesting close to conspecifics. There are many instances in which nest aggregations can be beneficial for the survival of ground‐breeding birds (Götmark and Andersson 1984; Hamilton 1971; Ringelman et al. 2014). When considering predation as the main source of mortality, birds breeding at higher nest densities can benefit from the vigilance of their neighbours warning of approaching predators. Although reeves only rarely alarm during incubation (Scheufler and Stiefel 1985), they often nest in assemblages that include very vocal sentinel birds such as northern lapwings, curlews ( *Numenius arquata* ) and black‐tailed godwits ( *Limosa limosa* ) (Belfín et al. 2024; Gochfeld 1984; personal observations). Furthermore, reeves flush from their nests upon detection of predators in the distance (Scheufler and Stiefel 1985; personal observations), which can signal to nearby incubating reeves potential predator presence or approaches. However, high nesting densities may also incur costs. Nest aggregations may attract more predators, which then prey on several nests that they encounter just by chance in the vicinity. Alternatively, predators may increase their search effort in areas where they have found a nest. Predators such as foxes are known to carry out targeted searches after identifying a nest (Seymour et al. 2003), thus increasing predation risk in denser nest clusters.

Daily nest mortality from predation tended to be lower for nests located closer to the meadow edges than for those further away across all models. This is contrary to our expectations based on a previous study, where we showed that reeves tended to nest further away from the meadow edges in some years, suggesting that they may avoid this area (Algora et al. 2025). Avoidance should lead to lower densities of nests closer to the edges, making nest search for predators in these areas less profitable. Similarly, curlews tend to avoid nesting close to woodlands, though their nest survival does not improve with distance from woodland habitats (Rivers et al. 2024). Many clutches close to the meadow edges may have been predated before we found them, or reeves may have already been breeding at a safe enough distance from the meadow edges. Furthermore, this pattern could also be explained by predator composition. In populations where foxes and avian species dwelling in forests are the main predators, edge effects would be expected to be stronger (Kaasiku et al. 2022). However, some avian predators such as marsh harriers ( *Circus aeruginosus* ), which we have regularly observed to prey on wader nests and broods in our study population (personal observations), hunt preferably in large open areas away from the forest edge (Upcott et al. 2024).

Daily nest survival differed within the season, with younger nests having higher daily nest survival probabilities than older nests. Nest success can show intraseasonal variation (e.g., Smith and Wilson 2010) due to nest age or changes in nest and predator density. Older nests typically present an increased exposure period to predation, decreasing their chances of survival. Furthermore, the observed pattern may also indicate variation of predator activity that is related to predators' reproductive seasons, as predators may forage on nests more when they require more energy to nourish their own young (e.g., Sieving and Willson 1999). The increase in sward height throughout the season may hinder predator detection and evasion, although it may also help to conceal clutches and incubating females. In addition, the clutches will also become more conspicuous with increasing development of the embryos. For example, many wader embryos vocalise close to hatching (Kostoglou et al. 2021), which will make it easier for predators to find the nests when they are nearby. Furthermore, parental behaviour can also change throughout the season as the reproductive value of nests increases (Smith and Wilson 2010). Incubating adults may carry out deceptive behaviours, such as nest defence or injury feigning, to increase nest survival from predation—though these can also increase predation threats on both adults and offspring (Gochfeld 1984; Montgomerie and Weatherhead 1988; Smith and Edwards 2018). Finally, it is also possible that shorter intervals in nest checks by researchers close to hatching attract predators, as researchers will leave fresh scent marks at every visit. To mitigate potential scent effects, we were careful to reduce steps in the immediate vicinity of the nest and laid false trails after each visit. However, we note that it remains unclear whether predators actually use human scent to find nests or human scent serves as deterrence (Weldon 2022).

In addition to the observed within‐year variation in nest mortality due to predation, we also observed variation between the years, with a moderate annual fluctuation in the strength of predation on nest survival. Part of this variation might be tied to changes in the availability of small mammals, the main prey of most predators, so in years when rodents are scarce, predation of eggs and chicks of ground‐nesting birds may increase (e.g., Pakanen et al. 2022). Yet, this compensation may not be enough to fully sustain predator populations resulting in fluctuation of predator and prey population abundances and thus contribute to the observed among‐year variation in wader nest predation (Pakanen et al. 2022). Factors affecting nest predation can also change annually in relation to nest age, as in the same coastal meadows, age‐specific nest survival of southern dunlins varied by year from negative to positive effects (see figure S6 in Pakanen et al. 2016).

### 4.3 Other Drivers of Nest Mortality

In addition to predation, the next main causes of ruff nest failure at our field site were abandonment, flooding and cow trampling. We assessed the effects of these other sources of mortality (including unknown causes) on daily nest survival probability in the *No‐predation* model. As in the other models, we found a quadratic effect of distance to other nests that favours intermediate distances to other nests, which in the *No‐predation* model could be explained by spatiotemporal autocorrelation of nest fates. This is because flooding and cow trampling, the main causes of failure accounted in this model, will have a strong impact on nest survival in certain parts of the study area, where they wipe out all (or most) nests at the same time including high density nest clusters. In contrast, when nests are more sparsely distributed across the area, the impact of local flooding or trampling will be less severe and affect only a few nests at a time.

In contrast to the other models, we found substantial seasonal variation in nest survival associated with laying date in the *No‐predation* model that included all nests. Clutches laid earlier in the season had higher daily survival chances than clutches laid later. We had previously shown that dunlin nest survival from flooding was lower in breeding seasons that started late, but we did not see such a temporal pattern in ruff nest survival at the time (Koivula et al. 2025). The impact of flooding on a breeding population can be lower when it happens early in the season, as birds can lay replacement clutches (Koivula et al. 2025; Pakanen et al. 2014). Furthermore, later dunlin nests have generally lower recruitment rates of chicks than earlier nests (Pakanen et al. 2016) and reeves typically nest a few weeks later than dunlins (Koivula et al. 2025). Late clutches will also have an elevated risk of trampling by cows as cattle are only released in early June when the first ruff clutches have hatched already.

### 4.4 Artificial Incubation of Clutches as a Method for Nest Protection

Overall, we found that artificially incubating clutches and substituting them with dummy eggs helped to reduce nest failure from some mortality sources. When only considering predation as a source of nest mortality (*Predation* model), ruff daily nest survival was higher in artificially incubated nests than in those without protection (Figure 2). This suggests that predators may be able to distinguish dummy eggs from natural ones, deterring them from foraging on the fake eggs. Similarly, in a study assessing nest predation in ground‐nesting birds, crows were able to perceive differences in plasticine and natural eggs, leading to lower predation rates of artificial clutches (Bravo et al. 2020).

Interestingly, artificial incubation was positively associated with mortality in the *No‐predation* model, with artificially incubated clutches having lower daily nest survival than the unprotected ones (Figure 2). We suggest that these results are an effect of the non‐random egg replacement that targeted high‐risk areas, since we did not design and implement a controlled experiment with proper control groups to assess the effectiveness of this intervention. Instead, we focused the intervention on saving high‐risk clutches that were near certain to have failed without intervention.

We artificially incubated the clutches of 45 nests (for over 24 h), out of which 19 clutches hatched thanks to our intervention (Table A1, Figure A2). Although only three of the nests protected against flooding for more than a day hatched, with the rest failing due to other sources of mortality, we also protected 16 other nests for less than 24 h, out of which six nests hatched successfully (Table A1, Figure A2). The fates of those briefly removed clutches did not influence the modelling results as they were not considered as artificially incubated nests in our models, but the short‐term treatment saved those clutches from certain flooding.

Artificial incubation was most effective in the clutches protected from cow trampling, as 16 out of 33 nests survived (Table A1, Figure A2). Nevertheless, the use of dummy eggs and artificial incubation was not fully effective, as we observed that cattle disturbance and/or dummy eggs rolling out of destroyed nest cups frequently led to nest abandonment (Table A1, Figure A2). Cow grazing is vital to prevent overgrowth and human‐induced accelerated natural succession in coastal marshes, which result in inadequate breeding habitat (Vehmaa et al. 2024). However, high grazing intensity and inadequate management can also lead to negative effects on breeding populations (Norris et al. 1998; Pakanen et al. 2011), especially through nest trampling (Beintema and Muskens 1987; Green 1988; Pakanen et al. 2011, 2016), which we show here is a major reason for nest failure in our population.

When considering the fates of only the naturally incubated nests, the effects of distance to paths and lay date on nest survival in the *No‐predation* model became weaker (Figure 3). This suggests that these effects were strongly influenced by the fate of the protected nests, as most of our interventions happened early to mid‐season coinciding with floods and cattle release for grazing. Similarly, nests closer to paths may survive trampling better and be more sheltered from floods, as most paths occur further away from the shoreline (also seen in the negative correlation between shoreline and paths, Figure A3F). We also note that the tendency of nests to survive better further away from the shoreline when not accounting for artificial incubation (Figure 3) disappears when the final fates of the nests with the dummy eggs are considered in the model (Figure 2), suggesting that the flooding intervention was effective in mitigating this risk.

### 4.5 Wader Decline, Management and Further Steps

Many European wader populations are steadily declining due to habitat degradation and low recruitment, and hence require extensive habitat management to maintain populations and reverse the declines (Laidlaw et al. 2017; Roodbergen et al. 2012). The modification of open landscapes, agricultural intensification and drainage of wetland ecosystems leads to the loss of wader breeding habitat (Kaasiku et al. 2019; Laidlaw et al. 2017; Pearce‐Higgins et al. 2017; Sutherland 2022; Wauchope et al. 2021). The remaining sites usually require management to sustain productive populations. Cow grazing is frequently used to prevent shrubification and maintain grassland habitats. However, as we show here, grazing during the nesting period can have a negative impact on nest survival and may contribute to low recruitment rates (Pakanen et al. 2011). Therefore, habitats maintained by grazing may in turn require adjusted grazing schedules or, if that's not possible, additional management such as targeted clutch collection and protection. Though laborious and not always successful, artificial incubation is a valuable method of clutch protection, especially in cases of emergency interventions where every nest counts. When most naturally incubated nests will fail, a comprehensive head‐starting program might be required to stabilise populations and provide critical time for other management actions to take effect. Head‐starting programs supply fledglings and hence skip the particularly vulnerable nesting and brood case stages (Donaldson et al. 2025). Further studies are needed to assess how cattle trampling impacts chick survival. Young shorebird chicks often use crouching for predator evasion, which is triggered when a threat approaches (Rohr‐Bender et al. 2024; Volkmer et al. 2024). This may make them particularly vulnerable to trampling by grazing cows.

Rapid changes in wetland habitats can lead to a mismatch between habitat selection cues and actual habitat quality, which can create ecological traps and sink habitats (Battin 2004; Kristan III 2003). Climate change is predicted to lead to higher occurrences of storm surges and wind floods due to increased frequency of thunderstorms (Gregow et al. 2020; Rädler et al. 2019; Vousdoukas et al. 2016). Although edge effects currently do not seem to play a major role for nest failure at Pitkänokka, they impact nest survival detrimentally in many other wader populations (Kaasiku et al. 2022) and could become a problem for local ruffs if birds seek refuge from increasing flooding risk and the nesting areas shift further away from the shore. Conversely, overgrowth of grasses and reeds in areas of the upper part of the meadow may push breeders towards the shoreline, where flooding risks are higher. Ruff nest and lek distributions have shifted throughout the years at Pitkänokka (Algora et al. 2025), but the reasons are not well understood. Studying the effects of vegetation, flooding and grazing on nest‐site selection and survival is needed at a higher spatial resolution and over longer time periods to design appropriate management practices and protect threatened wader populations.

## Author Contributions

**Hanna Algora:** conceptualization (lead), data curation (lead), formal analysis (lead), investigation (equal), methodology (equal), visualization (lead), writing – original draft (lead). **Krisztina Kupán:** formal analysis (supporting), methodology (equal), writing – review and editing (supporting). **Jelena Belojević:** investigation (supporting), writing – review and editing (equal). **Veli‐Matti Pakanen:** funding acquisition (equal), investigation (equal), writing – review and editing (supporting). **James D. M. Tolliver:** investigation (equal), writing – review and editing (supporting). **Veronika A. Rohr‐Bender:** investigation (supporting), writing – review and editing (supporting). **Kari Koivula:** conceptualization (equal), funding acquisition (equal), investigation (equal), supervision (equal), writing – review and editing (equal). **Clemens Küpper:** conceptualization (equal), funding acquisition (equal), investigation (equal), supervision (equal), writing – review and editing (equal).

## Funding

This work was supported by the Centre for Economic Development, Transport and the Environment of North Ostrobothnia, Ympäristöministeriö, PUTTE II, VN24452/2020, Tutkijakoulu, Oulun Yliopiston and Max‐Planck‐Gesellschaft.

## Conflicts of Interest

The authors declare no conflicts of interest.

## Data Availability

All data generated or analysed during this study are openly available in Zenodo at https://doi.org/10.5281/zenodo.15872587. The data set and scripts necessary for reproducing the analyses presented in this study are also included. Researchers and interested parties are encouraged to access and use these data in accordance with the terms of the Creative Commons Attribution 4.0 International licence.

## Appendix A

**TABLE A1:** Final nest fates for artificially incubated clutches based on the cause and duration of incubation.

Nest fates of artificially incubated clutches								
Protection from cattle trampling			Protection from flooding (> 24 h)			Protection from flooding (< 24 h)		
Fate	Freq	%	Fate	Freq	%	Fate	Freq	%
Hatched	16	48.5	Hatched	3	25.0	Hatched	6	37.5
Cattle trampled	10	30.3	Flooded	3	25.0	Predated	4	25.0
Abandoned	5	15.2	Predated	3	25.0	Flooded	3	18.8
Predated	1	3.0	Abandoned	3	25.0	Cattle trampled	1	6.3
Unknown fate	1	3.0				Abandoned	1	6.3
						Unknown dead	1	6.3

### Modifications to Original Preregistration

We published a preregistration of this study prior to the data analyses, in an effort to promote transparent research practices (Algora et al. 2024). Due to practical reasons regarding the analyses, we made modifications to our original methodology, which we summarise below. The nature of the changes did not alter our original hypotheses, although we outline the predictions linked to newly considered variables.

#### Modifications to Variables

We proposed studying the effect of nest density, but we continued using distance from nests to other nests to assess the effect of nesting in the vicinity of conspecifics, in order to be consistent with our previous projects. Our predictions remained the same despite the change in the variable.

We included the variable ‘artificial incubation’ to assess whether artificial incubation of clutches threatened by grazing or flooding could improve nest survival (including only the nests whose clutches were artificially incubated until hatching or nest failure). We predicted that nests would have lower survival upon the release of cattle in the meadows and when water levels rose more than 40 cm over sea level (Koivula et al. 2025). Consequently, we predicted that replacing threatened clutches with dummy eggs and artificial incubation would improve nest survival in all mortality scenarios.

We removed the variable ‘timing of breeding’ as it was similar to ‘lay date’ and added ‘nest age’ instead.

#### Modifications to Data Set

As we did not have reliable information on lek locations for 2016 and 2017, and the search effort in these years was variable, we decided to exclude these years from our final analyses.

Furthermore, given the complexity of our heterogeneous data including nests with artificially incubated clutches, we ran our analyses on two sets of data: (i) including nests with artificial incubation (*Survival of all nests*); and (ii) considering the fates of nests had we not intervened in their survival (*Survival of naturally incubated clutches*).

#### Modifications to Data Analysis Methodology

We carried out a better suited Bayesian approach to disentangle the drivers of nest mortality. Instead of the originally proposed method, we decided on the framework outlined by Korner‐Nievergelt et al. 2015 and described above, in which we had experience and which had more available supporting material.
